# Increased dynamin expression precedes proteinuria in glomerular disease

**DOI:** 10.1002/path.5181

**Published:** 2018-11-27

**Authors:** Ramzi Khalil, Klaas Koop, Reinhold Kreutz, Herman P Spaink, Pancras CW Hogendoorn, Jan A Bruijn, Hans J Baelde

**Affiliations:** ^1^ Department of Pathology Leiden University Medical Center Leiden The Netherlands; ^2^ Institute of Clinical Pharmacology and Toxicology, Charité – Universitätsmedizin Berlin, Humboldt‐Universität zu Berlin, and Berlin Institute of Health Berlin Germany; ^3^ Institute of Biology Leiden, Leiden University Leiden The Netherlands

**Keywords:** dynamin, proteinuria, kidney glomerulus, zebrafish, rats, histology

## Abstract

Dynamin plays an essential role in maintaining the structure and function of the glomerular filtration barrier. Specifically, dynamin regulates the actin cytoskeleton and the turnover of nephrin in podocytes, and knocking down dynamin expression causes proteinuria. Moreover, promoting dynamin oligomerization with Bis‐T‐23 restores podocyte function and reduces proteinuria in several animal models of chronic kidney disease. Thus, dynamin is a promising therapeutic target for treating chronic kidney disease. Here, we investigated the pathophysiological role of dynamin under proteinuric circumstances in a rat model and in humans. We found that glomerular *Dnm2* and *Dnm1* mRNA levels are increased prior to the onset of proteinuria in a rat model of spontaneous proteinuria. Also, in zebrafish embryos, we confirm that knocking down dynamin translation results in proteinuria. Finally, we show that the glomerular expression of dynamin and cathepsin L protein is increased in several human proteinuric kidney diseases. We propose that the increased expression of glomerular dynamin reflects an exhausted attempt to maintain and/or restore integrity of the glomerular filtration barrier. These results confirm that dynamin plays an important role in maintaining the glomerular filtration barrier, and they support the notion that dynamin is a promising therapeutic target in proteinuric kidney disease. © 2018 The Authors. *The Journal of Pathology* published by John Wiley & Sons Ltd on behalf of Pathological Society of Great Britain and Ireland.

## Introduction

Chronic kidney disease (CKD) is a major health issue worldwide 1. The progression of CKD is accompanied by a reduction in the glomerular filtration rate and subsequent proteinuria. In order to develop new therapeutic strategies for CKD, it is important to understand the mechanisms and processes that underlie glomerular filtration. The glomerular filtration barrier (GFB) consists of several components, including the interdigitating foot processes of podocytes, the glomerular basement membrane, and a glycocalyx‐covered fenestrated endothelium. Disrupting the GFB allows the passage of proteins into the urinary space. Under normal conditions, these proteins are then reabsorbed by proximal tubular epithelial cells; however, if the reabsorption mechanism is impaired or saturated, proteinuria can develop.

Dynamin is a recently identified protein that plays an important role in maintaining GFB integrity. This 96 kDa GTPase is expressed both in glomerular podocytes and in tubular epithelial cells 2, 3. Within the GFB, dynamin has three identified functions: (1) dynamin is involved in the turnover of nephrin 4; (2) it interacts directly with actin and actin‐regulatory proteins 5; and (3) it is involved in the endocytosis of albumin by podocytes 6. Several groups have reported that loss of dynamin using a genetic knockdown model or via cleavage with the endopeptidase cathepsin L results in proteinuria 3, 4, 7. Moreover, Schiffer *et al* demonstrated that dynamin is a potential therapeutic target in CKD 7. Specifically, the authors reported beneficial effects of treating several animal models of proteinuria with Bis‐T‐23, a small molecule compound that stimulates the oligomerization of dynamin to form a 72‐subunit helical structure 8. Importantly, Schiffer *et al* found that administering Bis‐T‐23 restored the ultrastructure of podocyte foot processes, decreased proteinuria, lowered mesangial collagen IV deposition, reduced mesangial matrix expansion, and prolonged survival 7. Ono *et al* also showed that Bis‐T‐23 treatment prevented albuminuria and attenuated alterations in foot process formation in an experimental mouse model 9. Although these studies indicate that dynamin plays an important role in maintaining GFB structure and function, the majority of this research relied on the observation of morphological and/or functional changes in the kidney after genetic manipulation or other alterations in dynamin. Thus, intrinsic changes in dynamin expression under proteinuric conditions have not been investigated in animal models or patients.

Here, we report that dynamin expression is increased in proteinuric conditions. We show that glomerular *Dnm2* and *Dnm1* mRNA levels are increased in a rat model of spontaneous proteinuria prior to the onset of proteinuria. We also show that knocking down dynamin translation in a zebrafish embryo model results in proteinuria. Lastly, we show that glomerular dynamin and cathepsin L protein levels are increased in human patients with proteinuric kidney disease.

## Materials and methods

### Microarray analysis

We analyzed microarray datasets of Dahl and SHR rats in order to identify differentially regulated cytoskeleton‐related genes. The datasets were retrieved from the Gene Expression Omnibus of the NCBI, which is accessible using the GEO Series accession number GSE 13810. This analysis was performed using Affymetrix GeneChip Rat Genome 230 2.0 arrays (Thermo Fisher Scientific, Waltham, MA, USA). Pathway analysis was performed using the Gene Ontology Tree Machine (Oak Ridge National Laboratory, Oak Ridge, TN, USA) and by global testing in the Kyoto Encyclopedia of Genes and Genomes (Kanehisa Laboratories, Kyoto, Japan) in order to determine which differentially regulated genes are involved in cytoskeletal regulation 10, 11. This microarray was performed on glomeruli obtained from animals at 4 and 6 weeks of age (Table 1).

**Table 1:** Differential glomerular expression of cytoskeleton‐related genes between 4‐ and 6‐week‐old Dahl rats and SHR rats

**Gene name**	Gene symbol	Chromosomal location	Fold‐difference in expression	Function
Periplakin	*Ppl*	10q12	4.17	Intermediate filament binding
Moesin	*Msn*	Xq31	3.86	Actin filament–membrane cross‐linking
Dynamin 1	*Dnm1*	3p11	3.36	Actin dynamics regulation
Tropomyosin 4	*Tpm4*	16p14	2.73	Actin binding
Thymoma viral proto‐oncogene 1	*Akt1*	6q32	2.71	Cell projection organization and biogenesis
Supervillin	*Svil*	17q12	2.62	Actin binding
Parvin, alpha	*Parva*	1q33	2.62	Actin cytoskeleton organization and biogenesis
Plastin 3 (T‐isoform)	*Pls3*	Xq14	2.51	Actin filament organization
Tropomyosin 1, alpha	*Tpm1*	8q24	2.23	Actin filament capping
Microtubule‐associated protein, RP/EB family, member 1	*Mapre1*	3q41	2.2	Regulation of microtubule polymerization
Rho guanine nucleotide exchange factor (GEF) 17	*Arhgef17*	1q32	2.1	Actin cytoskeleton organization and biogenesis
Caldesmon 1	*Cald1*	4q22	2.1	Actin binding
Signal‐regulatory protein alpha	*Sirpa*	3q36	2.04	Actin filament organization
WD repeat domain 44	*Wdr44*	Xq12	2	Vesicle recycling
Myosin Ib	*Myo1b*	9q22	1.99	Actin binding
Echinoderm microtubule‐associated protein‐like 4	*Eml4*	6q12	1.97	Microtubule stabilization
Actin‐related protein 2/3 complex, subunit 1B	*Arpc1b*	12p11	1.89	Cytoskeleton organization
Filamin, beta	*Flnb*	15p14	1.87	Actin binding
Myosin IC	*Myo1c*	10q24	1.78	Actin binding
Mitogen‐activated protein kinase 1	*Map3k1*	2q14	1.78	Actin filament polymerization
CAP, adenylate cyclase‐associated protein 1 (yeast)	*Cap1*	5q36	1.73	Actin cytoskeleton organization and biogenesis
Kinesin light chain 1	*Klc1*	6q32	1.59	Microtubule motor activity
Src homology 2 domain‐containing transforming protein C1	*Shc1*	2q34	1.57	Actin cytoskeleton organization and biogenesis
ARP1 actin‐related protein 1 homolog A (yeast)	*Actr1a*	1q54	1.55	Cytoskeleton organization
Actin, beta	*Actb*	12p11	1.54	Cytoskeleton organization
A kinase (PRKA) anchor protein 2	*Akap2*	5q24	1.51	Actin filament organization
Spectrin alpha 1	*Spna1*	13q24	0.19	Cytoskeleton organization
Polyamine modulated factor 1 binding protein 1	*Pmfbp1*	19q12	0.14	Cytoskeleton organization and biogenesis

The micro array data analyzed in this study have been deposited in NCBI's Gene Expression Omnibus and are accessible through GEO Series accession number GSE 13810 (https://www.ncbi.nlm.nih.gov/geo/query/acc.cgi?acc=GSE13810).

### Animal studies: rats

The rat experiments were approved by the Institutional Ethics Committee for Animal Care and Experimentation. Male spontaneously proteinuric Dahl salt‐sensitive rats (Dahl) and non‐proteinuric spontaneously hypertensive rats (SHR) were obtained from the Charité‐University Medicine Berlin 12. To prevent the early accelerated development of severe hypertension, the rats were fed a low‐salt diet containing 0.2% NaCl by weight content. Tissues were collected from animals at 2, 4, 6, 8, and 10 weeks of age. A total of 67 animals were used, with respectively seven, seven, seven, eight, and eight Dahl rats per age group and five, four, six, seven, and eight SHR rats per age group. In each animal, the left kidney was used to isolate the glomeruli using magnetic retraction; these samples were used for mRNA analysis 13. The right kidney of each animal was extracted; part was embedded in paraffin wax, and another part was snap‐frozen and stored −80°C.

### RNA isolation, reverse transcription, and qPCR

RNA was isolated from rat glomeruli using TRIzol (Invitrogen, Waltham, MA, USA). AMV reverse transcriptase (Roche Diagnostics, Risch‐Rotkreuz, Switzerland) was used to reverse‐transcribe the RNA into first‐strand cDNA, which was then analyzed using qPCR with the primer pairs listed in Table 2. *Hprt1* was used as an internal control. qPCR was performed on an iCycler real‐time PCR machine with SYBR Green Supermix (Bio‐Rad Laboratories, Hercules, CA, USA), and iCycler IQ 3.1 software (Bio‐Rad Laboratories) was used to analyze gene expression and to normalize the data. *Dnm1* and *Dnm2* mRNA levels are shown as relative to the average *Dnm1* or *Dnm2* mRNA expression in SHR rats.

**Table 2:** Primer sequences used for RT‐PCR analysis

Name (species)	Gene symbol	mRNA accession number	Forward primer	Reverse primer
Dynamin 1 (rat)	*Dnm1*	NM_080689.4	TTGATGAGAAGGAACTGCGAAGG	AAGCGAGGTCAGGAGTGAAGAG
Dynamin 2 (rat)	*Dnm2*	NM_013199.1	TGAAATGCGTGGACCTGGTT	CAATGCGTTCGGTCTCCTCT
Cathepsin L (rat)	*Ctsl*	NM_013156.2	CAGTGGAAGTCCACACACAGA	GTGCTTCCCGTTGCTGTACT
Hypoxanthine phosphoribosyltransferase 1 (rat)	*Hprt1*	NM_012583.2	GGCTATAAGTTCTTTGCTGACCTG	AACTTTTATGTCCCCCGTTGA

### Animal studies: zebrafish

All zebrafish experiments were performed prior to the free‐feeding embryo stage and are therefore not considered animal experiments in accordance with the EU Animal Protection Directive 2010/63/EU. Wild‐type (WT) AB/TL strain zebrafish (*Danio rerio* H) were maintained using previously defined standards 14. Embryos were obtained through natural crossings and kept in E3 medium at 28.5°C. In the one‐ to four‐cell stage, the embryos were injected with 1 nl of a morpholino (Gene Tools, Philomath, OR, USA) targeting the *dnm2* gene (to knockdown mRNA translation) or a scrambled control morpholino 15.

### Glomerular permeability and tubular reabsorption assay

A previously validated tubular dextran reabsorption model was used to assess glomerular permeability in zebrafish embryos 16, 17. At 4 days post‐fertilization (dpf), a group of WT embryos were injected with 3 nl of puromycin aminonucleoside (PAN, 25 mg/ml; Sigma‐Aldrich, St Louis, MO, USA) to induce kidney damage as a positive control for proteinuria 17. At 5 dpf, all groups were injected with 3 nl of a mixture of TRITC‐labeled 3 kDa dextran (100 mg/ml; Invitrogen) and FITC‐labeled 70‐kDa dextran (25 mg/ml; Invitrogen). One hour after injection, the animals were fixed in 10% formalin for 24 h. After subsequent storage in 70% ethanol, the embryos were embedded in paraffin, sectioned at 3 μm thickness, and examined using fluorescence microscopy for the presence of reabsorption droplets in the proximal tubule epithelial cells by an investigator who was blinded with respect to the treatment conditions. The number of reabsorption droplets in the proximal tubule cells was counted and compared between groups. The 3 kDa dextran tracer freely passes the GFB and is subsequently reabsorbed by the proximal tubule epithelial cells. Therefore, reabsorption of this tracer is used as an indicator of sufficient tubular reabsorption function. In contrast, the 70 kDa tracer does not readily pass the GFB under normal conditions and is therefore used as an indicator of glomerular permeability 16. The PAN and dextran injections were performed under anesthesia with 4% tricaine methanesulfonate.

### Human materials

Biopsies from 26 patients with a variety of proteinuric kidney diseases were obtained from the LUMC tissue archive. Eight biopsies were from patients with minimal change disease; three were from patients with focal segmental glomerulosclerosis; three were from patients with IgA nephropathy; six were from patients with lupus nephritis; and six were from patients with diabetic nephropathy. In addition, 16 samples were used as a control group; these samples consisted of biopsy material obtained from patients with no evidence of glomerular pathology whose kidneys were unsuitable for transplantation due to technical reasons, or from autopsy tissue. Patient characteristics are summarized in Table 3. All tissue samples were obtained and handled in accordance with institutional guidelines and with the Code of Conduct regarding the responsible use of human tissues 18.

**Table 3:** Patient characteristics

	Control	DN	LN	MCD	FSGS	IgAN
Number of patients (*n*)	15	6	6	8	5	4
Mean age, years (SD)	54.8 (16.11)	50.2 (19.11)	29.3 (12.15)	31.1 (20.88)	36.2 (7.19)	34.5
Mean serum creatinine, μmol/l (SD)	101.57 (37.95)	140.80 (76.30)	112.80 (39.51)	77.38 (27.77)	112.80 (60.24)	107.5 (34.09)
Proteinuria, g/24 h (SD)	0.14 (0.12)	1.15 (0.82)	2.50 (1.86)	3.56 (3.32)	5.08 (1.84)	4.40 (3.19)

### Immunohistochemistry

The mouse anti‐dynamin antibody Hudy 1 (Upstate Biotechnology, Lake Placid, NY, USA; catalog number 05‐319, diluted 1:80) was used to detect dynamin. Hudy 1 is a monoclonal antibody that recognizes both dynamin 1 and dynamin 2. Hudy 1 recognizes the epitope of residues 822–838 within the proline‐rich domain of dynamin. Anti‐mouse Envision (Dako Cytomotion, Glostrup, Denmark) was used as a secondary antibody to detect the primary antibody. A rabbit anti‐cathepsin L antibody (Abcam, Cambridge, UK; ab203028, diluted 1:100) was used to detect cathepsin L. The results were analyzed by measuring the percent positive area in the glomeruli using the ImageJ digital image analysis program. No scarred glomeruli were included in these analyses.

### Statistical analyses

Statistical analyses were performed using SPSS version 23.0 (IBM Corp, Armonk, NY, USA). Levels of mRNA and protein were compared using one‐way ANOVA (with Dunnett's *post hoc* analysis when more than three groups were compared). Student's unpaired *t*‐test was used when two or three groups were compared. Correlation analysis was performed using the Pearson correlation coefficient. Differences with a *P* value less than 0.05 were considered significant.

## Results

### Gene expression profiling between Dahl and SHR rats

First, we investigated differences in the gene expression between Dahl rats and SHR rats using microarray analysis. With respect to cytoskeleton‐related genes, we found 28 genes that were differentially regulated between Dahl rats and SHR rats (Table 1). Of these 28 genes, 26 were significantly upregulated (including *Dnm1*), and two genes (*Spna1* and *Pmfbp1*) were downregulated.

### Glomerular *Dnm1* and *Dnm2* mRNA – but not dynamin protein area expression – are increased in Dahl rats prior to the onset of proteinuria

**Figure 1:**
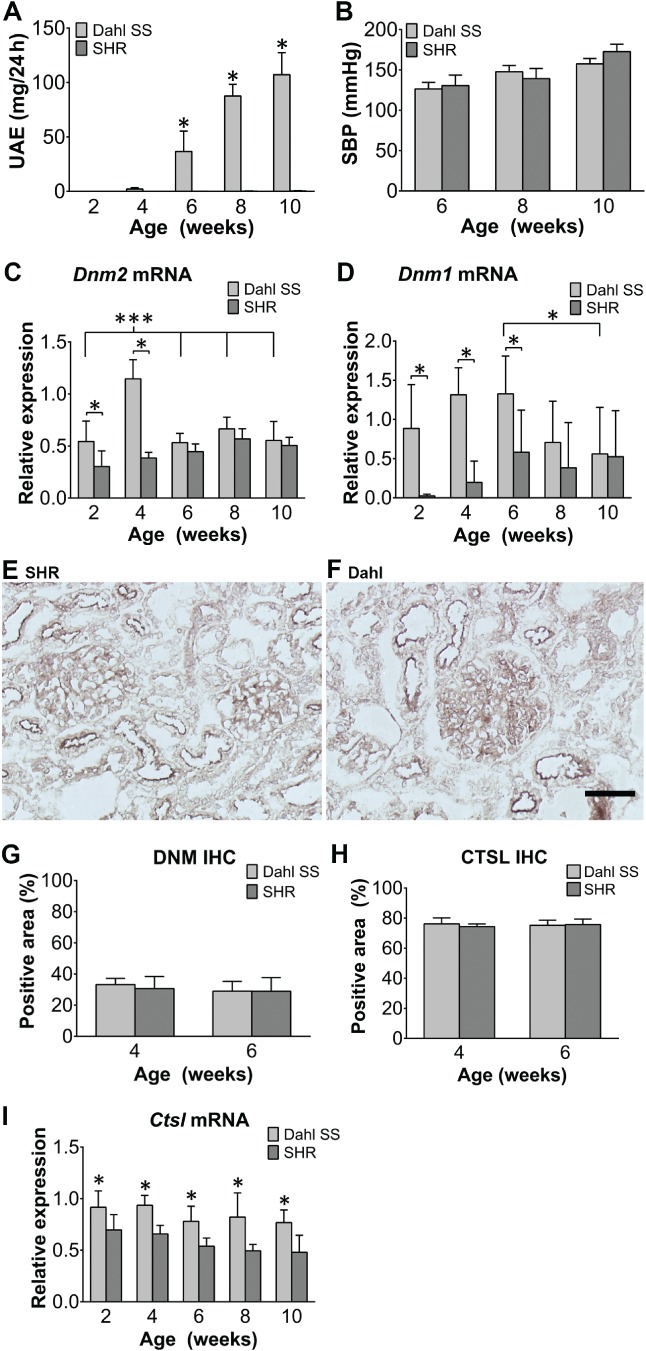
Glomerular *Dnm2 and Dnm1* mRNA levels – but not dynamin protein levels – are increased in Dahl rats prior to the onset of proteinuria. (A, B) Summary of urinary albumin excretion (UAE) (A) and systolic blood pressure (SBP) (B). (C, D) Summary of glomerular *Dnm2* mRNA (C) and *Dnm1* mRNA (D). (E, F) Example images of glomeruli from an SHR rat (E) and a Dahl rat (F) immunostained for dynamin. Similar to the pattern seen in human kidney sections, dynamin protein was present in podocytes and the tubular brush border. (G–I) Summary of the percent glomerular positive area for dynamin protein (G), the percent glomerular positive area for cathepsin L protein (H), and glomerular cathepsin L (*Ctsl*) mRNA (I) in Dahl and SHR rats at the indicated ages. No difference was seen for either dynamin or cathepsin L percent glomerular positive area. *p < 0.05 versus the SHR group. The images in E and F were taken at the same magnification. Scale bar = 50 μm.

Next, we measured glomerular dynamin expression in the Dahl salt‐sensitive spontaneously proteinuric and hypertensive rat model (referred to here as simply Dahl rats) and the spontaneously hypertensive rat (SHR). Dahl rats under a low dietary salt intake developed significant proteinuria beginning at 6 weeks of age; in contrast, SHR rats did not develop proteinuria, even at 10 weeks of age (Figure 1A). Consistent with the spontaneous hypertension phenotype, both strains developed hypertension to a similar extent beginning at 8 weeks of age (Figure 1B).

As *Dnm2* is the ubiquitous and prevalent form of dynamin, we measured both glomerular *Dnm2* and *Dnm1* mRNA levels (Figure 1C,D, respectively). When analyzing only the Dahl group, no significant differences in glomerular *Dnm2* mRNA levels were seen between weeks 2, 6, 8, and 10. However, these levels were higher at 4 weeks of age than at all other time points (*p* < 0.0001 compared with weeks 2, 6, 8, and 10; Figure 1C). In SHR rats, a significant difference in glomerular *Dnm2* levels was found when week 2 was compared with weeks 8 and 10 (*p* < 0.001 and *p* < 0.01, respectively) and also between weeks 4 and 8 (*p* < 0.05, Figure 1C). A similar analysis of glomerular *Dnm1* mRNA levels in Dahl rats showed a gradual increase from week 2 to week 6, after which the levels decreased. This difference is significant between weeks 6 and 10 (*p* < 0.05, Figure 1D). SHR rats showed very low expression of glomerular *Dnm1* mRNA at week 2 which increased slightly, but not significantly, up until 6 weeks (Figure 1D). None of the differences in mRNA levels at the different time points were significant in this group. When comparing dynamin mRNA levels between Dahl and SHR rats, the level of glomerular *Dnm2* mRNA was significantly increased in Dahl rats compared with SHR rats at 2 and 4 weeks of age (Figure 1C, *p* < 0.05 and *p* < 0.0001, respectively). Glomerular *Dnm1* mRNA was significantly higher in Dahl rats than in SHR rats at 2, 4, and 6 weeks of age (Figure 1D). Interestingly, the levels of both *Dnm1* mRNA and *Dnm2* mRNA at 2 weeks of age were higher in Dahl rats than in SHR rats, which is before the onset of proteinuria (i.e. at 6 weeks of age). Also, when comparing the different age groups, animals that will become proteinuric (i.e. the Dahl rats) have an increase in glomerular *Dnm2* expression prior to the onset of proteinuria. When comparing PCR *C*_t_ values, *Dnm2* mRNA levels were found to be more than ten times higher than *Dnm1* levels. With respect to dynamin protein levels, immunohistochemistry revealed that the protein was present at the tubular brush border and in podocytes in both Dahl and SHR rats (Figure 1E,F) at similar levels at both 4 and 6 weeks of age (i.e. after Dahl rats develop proteinuria) (*p* = 0.6, Figure 1G).

### *Ctsl* mRNA levels – but not protein area expression – are higher in Dahl rats than in SHR rats

Cathepsin L, encoded by the *Ctsl* gene, is an enzyme that cleaves dynamin 3. We therefore measured the expression of cathepsin L in the glomeruli of Dahl and SHR rats using RT‐PCR (Figure 1I). We found that *Ctsl* mRNA levels were significantly higher in the glomeruli of Dahl rats compared with SHR, ranging from 1.3‐fold to 1.7‐fold higher, at all ages tested (*p* < 0.01). Immunohistochemistry revealed that there was no difference between Dahl and SHR rats in the glomerular cathepsin L‐positive area (*p* = 0.11 and *p* = 0.70 at 4 and 6 weeks of age, respectively; Figure 1H).

### Knocking down dynamin translation causes proteinuria

**Figure 2:**
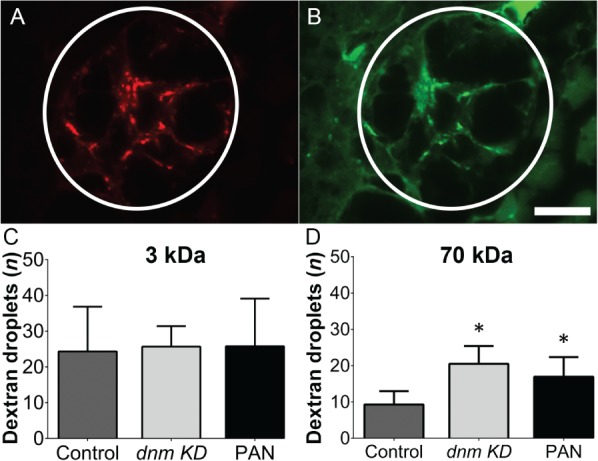
Blocking the translation of *dnm* mRNA in zebrafish embryos causes proteinuria. Wild‐type zebrafish embryos were injected with an anti‐*dnm* morpholino or a scrambled control morpholino, followed by a mixture of 3 and 70 kDa dextran molecules. (A, B) Representative fluorescence images of the proximal tubule epithelial cells (circled structure) in a dynamin‐knockdown zebrafish embryo. (C, D) The number of fluorescent droplets below the luminal surface was counted in control embryos, dynamin‐knockdown embryos, and wild‐type embryos injected with puromycin aminonucleoside (PAN) as a positive control for increased glomerular permeability. *p < 0.05 versus control. A digital high‐pass filter has been placed over panels A and B to enhance the contrast between reabsorption droplets and the surrounding tissue. The images in A and B were taken at the same magnification. Scale bar = 10 μm.

Next, we knocked down dynamin expression in zebrafish embryos using morpholino injection followed by injection of a mixture of 3 and 70 kDa dextran tracers (Figure 2A,B). As a positive control for inducing proteinuria, a separate group of embryos received an injection of puromycin aminonucleoside (PAN). We then quantified the reabsorption of dextran droplets. Our analysis revealed that the mean number of 3 kDa dextran droplets was similar between control zebrafish (which received an injection of a scrambled morpholino), dynamin‐knockdown zebrafish, and PAN‐injected zebrafish (*p* = 0.96, Figure 2C), indicating that knockdown of dynamin in this model does not significantly affect tubular reabsorption. In contrast, the dynamin‐knockdown zebrafish had significantly more reabsorption of 70 kDa droplets compared with control zebrafish (*p* < 0.0001, Figure 2D), indicating that loss of dynamin increases glomerular permeability, as also shown in the zebrafish model by Schiffer *et al* 7.

### Dynamin protein levels are increased in the glomeruli of patients with proteinuria

**Figure 3:**
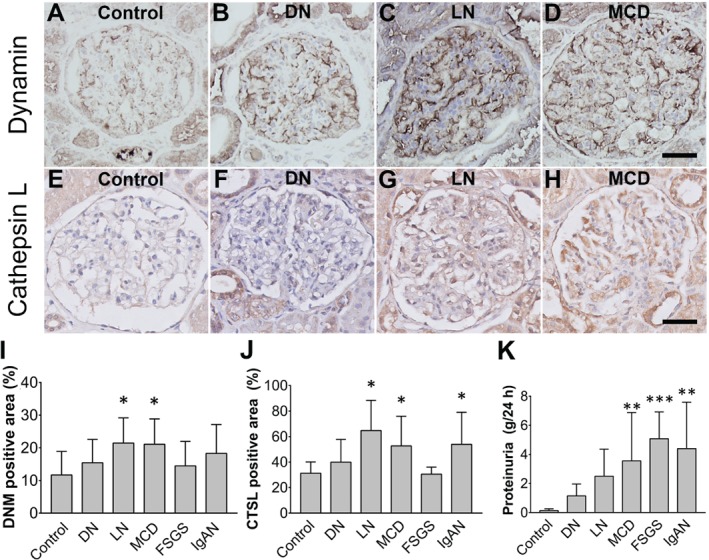
Glomerular dynamin protein levels are increased in human proteinuric kidneys. (A–H) Representative images of glomeruli immunostained for dynamin (A–D) or cathepsin L (E–H) in a healthy control subject (A, E), a patient with diabetic nephropathy (B, F), a patient with lupus nephritis (C, G), and a patient with minimal change disease (D, H). (I, J) Summary of the percent glomerular positive area for dynamin (I) and cathepsin L (J) in patients with the indicated proteinuric kidney diseases. Dynamin protein expression is significantly higher in LN and MCD compared with control; *p < 0.05 versus control. Cathepsin L protein expression is significantly higher in LN, MCD, and IgAN compared with control; *p < 0.05 versus control. (K) Summary of the proteinuria data of the different patient groups. No correlation was found between the level of proteinuria and dynamin staining. Patients with MCD, FSGS, and IgAN all had significantly more proteinuria than control patients; **p < 0.01, ***p < 0.001. MCD, minimal change disease; FSGS, focal segmental glomerulosclerosis; IgAN, IgA nephropathy; LN, lupus nephritis; DN, diabetic nephropathy. All images were taken at the same magnification. Scale bar = 50 μm.

Finally, we examined the expression of dynamin in the kidneys of patients with proteinuria (Figure 3A–D). Biopsies were obtained from patients with proteinuric kidney disease and control subjects, and stained by immunohistochemistry for the presence of dynamin. Positive staining was found primarily in podocytes, at the tubular epithelial brush border, and in endothelial cells of larger vessels. When individual disease groups were compared with the controls, the glomeruli of patients with minimal change disease (MCD) and lupus nephritis (LN) had a significantly higher dynamin‐positive area percentage (*p* = 0.03 and *p* = 0.04, respectively; Figure 3I). No scarred glomeruli were included in these analyses. No correlation was found between proteinuria levels and dynamin expression levels (Figure 3K).

### Cathepsin L protein levels are also increased in the glomeruli of patients with proteinuria

The same cohort was also stained by immunohistochemistry for the presence of cathepsin L (Figure 3E–H). We found a significantly higher glomerular cathepsin L‐positive area percentage in patients with MCD, LN, and IgA nephropathy (IgAN; *p* < 0.0001 compared with controls in all three groups, Figure 3J). A positive correlation was found between the glomerular positive area percentages for dynamin and cathepsin L (*r* = 0.928, *p* = 0.0075).

## Discussion

Here, we have investigated dynamin expression under proteinuric conditions in both patients and rats. In addition, we examined whether dynamin is involved in glomerular proteinuria, tubular proteinuria, or both. We found a significant increase in the glomerular levels of *Dnm2* and *Dnm1* mRNA in Dahl rats prior to the onset of albuminuria. We also showed that knocking down dynamin translation results in proteinuria in a zebrafish embryo model. Lastly, we found that the glomerular levels of dynamin and cathepsin L protein are significantly increased in patients with proteinuric kidney disease.

Our finding of increased dynamin protein and *Dnm1* and *Dnm2* mRNA levels in proteinuric disease, combined with the results of Soda *et al* 4 showing that dynamin knock out results in proteinuria, leads to the following two hypotheses on dynamin's role in GFB integrity. First, a minimum level of dynamin may be required for adequate GFB integrity; thus, if this level is not reached, GFB integrity is lost. Second, when the GFB is under stress, dynamin may be upregulated in an attempt to maintain or restore GFB integrity. We previously studied the expression of proteins required for proper GFB function in patients with acquired proteinuria and found changes that suggest a compensatory mechanism 19. Thus, disease progression may occur when this compensatory mechanism becomes saturated or exhausted.

**Figure 4:**
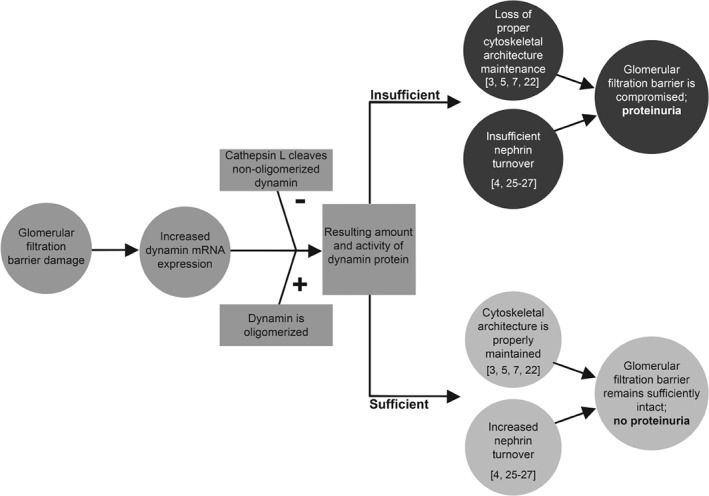
Increased dynamin expression precedes proteinuria. Flow chart illustrating the proposed compensatory mechanism in response to impaired integrity of the glomerular filtration barrier, in which increased dynamin expression precedes proteinuria. After damage to the glomerular filtration barrier, *DNM1* and *DNM2* are upregulated, leading to increased levels of *DNM1* and *DNM2* mRNA. Whether this increase results in increased levels of dynamin protein depends on the oligomerization status of dynamin and the activity of cathepsin L, which selectively cleaves non‐oligomerized dynamin 3. If the total amount of dynamin is sufficient, the glomerular filtration barrier remains intact, preventing the onset of proteinuria. However, if dynamin levels are insufficient – and/or if this compensatory response is exhausted – proteinuria develops. The suggested mechanisms of cytoskeletal architecture maintenance and nephrin turnover have been reported by others 3, 4, 5, 7, 8, 22, 23, 24.

In Dahl rats, which spontaneously develop proteinuria, we found that the levels of *Dnm1* and *Dnm2* mRNA are increased prior to the onset of proteinuria, a symptom that manifests only when protein is present in excreted urine. Proteinuria occurs when proteins pass through the GFB and are not sufficiently reabsorbed by the tubular system, due to either saturation or malfunction of the tubular reabsorption process. Thus, the increase in glomerular *Dnm1* and *Dnm2* mRNA levels prior to the onset of proteinuria might reflect a compensatory mechanism in response to an early, presymptomatic increase in GFB permeability that does not yet lead to actual proteinuria. However, as proteinuric disease progresses, this compensatory system can become exhausted, thereby failing to prevent the onset of proteinuria. This hypothetical process is depicted schematically in Figure 4.

Interestingly, we found that while animals that will become proteinuric (Dahl rats) express both more glomerular *Dnm2* and *Dnm1* mRNA before the onset of proteinuria, this does not result in an increase in dynamin protein. However, since dynamin is a regulatory GTPase, it is not necessary to have an increase in protein level to have an altered intracellular activity. Also, cathepsin L‐mediated cleavage of dynamin may be increased and cause a decrease in dynamin protein staining 3. In humans, we found that there is indeed an increase in glomerular cathepsin L protein which directly and strongly correlated with glomerular dynamin protein levels, as also observed by Sever *et al* 3. Cleavage of dynamin by cytosolic L generates a 40 kDa N‐terminal fragment of dynamin. As the used antibody recognizes an epitope of residues 822–838 of dynamin, these fragments were not identifiable in the immunohistochemistry experiments performed in this study. In our study, we found significantly higher levels of *Ctsl* mRNA in Dahl rats at all ages investigated, suggesting that in this rat model both the transcription and the post‐translational cleavage of dynamin are increased. Although the Hudy 1 antibody used does not distinguish between DNM1 and DNM2 proteins, the higher *Dnm2* mRNA levels suggest that *Dnm2* is the more prevalent type of dynamin, as also reported by others 4.

We found that the glomerular levels of dynamin protein were increased primarily in patients with minimal change disease (MCD) and lupus nephritis. Schiffer *et al* recently proposed that dynamin's principal role in maintaining podocyte structure and preventing proteinuria is independent of the underlying disease pathogenesis 7 Our results are consistent with this notion, given that lupus nephritis is considered to be immunological in origin, whereas MCD is not 20, 21.

Sever *et al* previously reported that the levels of *CTSL* mRNA were increased in the glomeruli of patients with acquired proteinuric disease 3. Notably, the levels of *CTSL* mRNA expression were increased in patients with focal segmental glomerulosclerosis (FSGS) and in patients with diabetic nephropathy, but not in patients with MCD. Interestingly, we found that dynamin expression was significantly increased in patients with MCD, but not in patients with FSGS or diabetic nephropathy; Sever *et al* did not measure *CTSL* mRNA in patients with lupus nephritis. In our study, we found significantly more cathepsin L protein in the glomeruli of patients with MCD, lupus nephritis, and IgA nephropathy. Although we did see an increase in the glomerular cathepsin L‐positive area percentage in DN patients, as also reported by Sever *et al*, this increase was not statistically significant.

Thus, taken together, these results suggest that in human proteinuric disease, the interplay between cathepsin L and dynamin is part of a regulatory system influenced by proteinuria, as proposed previously 3.

Dynamin's protective effect on the GFB could be effected through its role in nephrin turnover, as suggested by other groups 4, 22, 23, 24. Nephrin (encoded in rats by the *Nphs1* gene) is a core component of the glomerular slit diaphragm 25. In podocytes, nephrin is internalized by clathrin‐mediated endocytosis and clathrin‐independent raft‐mediated endocytosis, both of which are dynamin‐dependent processes 23. We previously reported that *Nphs1* mRNA levels are increased significantly in Dahl rats by 10 weeks of age, although the pattern of nephrin protein is focal and segmental rather than linear, and effacement of the podocyte foot process occurs 26. In this study, we found that the levels of *Dnm1* and *Dnm2* mRNA are significantly lower at 10 weeks of age compared with younger ages. These results suggest that the relative decrease in *Dnm1* and *Dnm2* mRNA in Dahl rats at later ages results in a loss of sufficient nephrin turnover, which then leads to reduced protection against podocyte pathology.

Dynamin also directly interacts with the actin cytoskeleton, as shown by others 3, 5, 7, 8. Consistent with this structural role, our microarray analysis revealed additional evidence that the cytoskeletal architecture is disrupted in Dahl rats, given the differential regulation of cytoskeleton‐related genes. Further studies will likely provide new insight into how the actin cytoskeleton is regulated under proteinuric conditions.

In conclusion, our results provide evidence that dynamin expression is increased in human proteinuric disease. Moreover, we propose that a minimum level of dynamin is required for GFB integrity, and increases in *Dnm1* and *Dnm2* mRNA are part of a compensatory mechanism that supports GFB integrity under stressful conditions. Given that this mechanism seems to play a role in patients with proteinuric kidney disease, dynamin may represent a promising target for therapeutic intervention.

## Author contributions statement

RKh designed and performed experiments, analyzed data, and wrote the paper. KK designed and performed experiments. HPS, RKr, PCWH, and JAB provided conceptual advice. HJB designed experiments and provided technical support and conceptual advice. All authors had final approval of the submitted and published versions.
